# Efficacy and safety of intracoronary prourokinase during percutaneous coronary intervention in treating ST-segment elevation myocardial infarction patients: a randomized, controlled study

**DOI:** 10.1186/s12872-020-01584-0

**Published:** 2020-06-26

**Authors:** Yanqiang Wu, Xianghua Fu, Qiang Feng, Xinshun Gu, Guozhen Hao, Weize Fan, Yunfa Jiang

**Affiliations:** 1 Department of Cardiology, The Second Hospital of Hebei Medical University, 215 West Heping Road, Shijiazhuang, 050000 China; 2 Department of Cardiology, Handan Central Hospital, Handan, 056000 China

**Keywords:** STEMI, PCI, Prourokinase, Efficacy, Safety

## Abstract

**Background:**

Prourokinase is a single-chain plasminogen activator presenting with fewer hemorrhagic complications and reduced reocclusion rate compared with the conventional fibrinolytic agents in patients with coronary artery disease. However, prourokinase intracoronary injection during PCI for treating patients with ST-segment elevation myocardial infarction (STEMI) is rarely investigated. Therefore, this study aimed to evaluate the efficacy and safety of intracoronary prourokinase during the percutaneous coronary intervention (PCI) in treating STEMI patients.

**Methods:**

Fifty STEMI patients who underwent primary PCI were consecutively enrolled and randomly assigned to intracoronary prourokinase group (*N* = 25) or control group (*N* = 25). During the primary PCI procedure, patients in the intracoronary prourokinase group received 10 ml prourokinase injection, while patients in control group received 10 ml saline injection as control. The primary endpoints were coronary physiological indexes, the secondary endpoints were angiographic assessments, myocardial infarct size/reperfusion assessment, cardiac function evaluations, major adverse coronary events (MACEs) and hemorrhagic complications. All patients were followed up for 3 months.

**Results:**

Post PCI, the index of microcirculatory resistance (IMR) was decreased in intracoronary prourokinase group than that in control group (34.56 ± 7.48 vs. 49.00 ± 8.98, *P* < 0.001), while no difference of coronary flow reserve (CFR) (2.01 ± 0.32 vs. 1.88 ± 0.23, *P* = 0.267) or fractional flow reserve (FFR) (0.89 ± 0.05 vs. 0.87 ± 0.04, *P* = 0.121) was found between the two groups. The thrombolysis in myocardial infarction myocardial perfusion grade (TMPG) (*P* = 0.024), peak values of creatine kinase (CK) (*P* = 0.028), CK isoenzyme-MB (CK-MB) (*P* = 0.016), cardiac troponin I (cTnI) (*P* = 0.032) and complete ST-segment resolution (STR) (*P* = 0.005) were better in intracoronary prourokinase group compared with control group. At 3-months post PCI, left ventricular ejection fraction (LVEF) and wall motion score index (WMSI) were higher, while left ventricular end-diastolic diameter (LVEDd) was lower in intracoronary prourokinase group compared with control group (all *P* < 0.05). There was no difference in hemorrhagic complication or total MACE between the two groups.

**Conclusion:**

Intracoronary prourokinase during PCI is more efficient and equally tolerant compared with PCI alone in treating STEMI patients.

**Trial registration:**

Chinese Clinical Trial Registry ChiCTR1800016207. Prospectively registered.

## Background

ST-segment elevation myocardial infarction (STEMI), united by persistent ischemic chest pain over 20 min, the ST-segment elevation and the increase of myocardial necrosis biomarkers, is still a rather prevalent cause of mortality and morbidity in clinical practice [1]. Percutaneous coronary intervention (PCI) has evolved as the mainstay of STEMI management since its first introduction four decades ago, and with the development of new technology, patients’ prognosis has been primarily advanced [2]. However, apart from all the benefits that PCI brings to the patients attacked by STEMI, there also exist hemorrhagic and cardiovascular events, not to mention that in patients with a favorable outcome after PCI, a considerable proportion of 30-day and 1-year mortality still could be seen [3]. Therefore, more efforts must be made in the context of PCI treatment in STEMI patients.

Fibrin specific thrombolytic agents have been extensively used for thrombolysis in patients with STEMI, among which prourokinase is a single-chain plasminogen activator presenting with fewer hemorrhagic complications and reduced reocclusion rate in patients compared with the conventional fibrinolytic agents [4, 5]. Although very promising, prourokinase is still not a frequent drug used for STEMI patients due to the lack of evidence. To be specific, there are very limited studies investigating efficacy and safety of prourokinase in STEMI, among which, a large randomized clinical trial states that 20 mg prourokinase intravenously injection followed by 30 mg prourokinase intravenous infusion within 30 min is effective and tolerable in patients with STEMI [6]. Thus, we presumed that prourokinase might also be efficient and safe during PCI in treating patients with STEMI.

In addition, not only macrovascular but also microvascular is critical in the coronary disease, and the latter has been demonstrated to be even more crucial, hence, the indexes indicating coronary flow determined by the microvascular function, including index of microcirculatory resistance (IMR), coronary flow reserve (CFR) and fractional flow reserve (FFR) are important for assessing the function recovery of patients post PCI procedure [7, 8]. Besides, the assessment of indexes indicating angiographic status, myocardial infarct size, myocardial reperfusion and cardiac function are critical for the management of patients post PCI as well.

Therefore, this study evaluated the efficacy and safety of intracoronary prourokinase during PCI in treating STEMI by assessing the indexes indicating cardiac function and adverse events, respectively.

## Methods

### Patients

Between June 2017 and December 2017, 50 STEMI patients underwent primary PCI in the Second Hospital of Hebei Medical University were consecutively enrolled in the present study. Inclusion criteria were as follows: (1) persistent chest pain more than 30 min, and ST-segment elevation with the cut-off J points ≥ 1 mm in standard leads or ≥ 2 mm in contiguous precordial leads on electrocardiograph (ECG); (2) progressive ischemic chest pain or unstable hemodynamics with ST-segment elevating when the onset time reached 12 ~ 24 h; (3) age more than 18 years; (4) symptom onset within 6 h before randomization, and intention to undergo primary PCI. Patients were excluded if they presented with (1) suspected aortic dissection, infective endocarditis, significant valvular heart disease, or cardiac shock, (2) hematologic diseases, hemorrhagic diseases or bleeding propensity, (3) history of cerebral hemorrhage, subarachnoid hemorrhage, intracranial tumors, cerebral vascular malformations, other stroke or cerebrovascular events known within 3 months, (4) history of bleeding, trauma and visceral surgery within 4 weeks, (5) previous coronary artery bypass grafting (CABG), (6) severe head closed trauma or facial trauma within 3 months, (7) severe liver and kidney dysfunction, malignant tumor, acute inflammatory diseases, (8) pregnant women, (9) contraindications to prourokinase, aspirin, ticagrelor and heparin. Besides, angiographic exclusions included the left main disease with stenosis more than 50%, no obvious thrombus burden, severe coronary tortuosity or significant distal stenosis, and chronic total occlusion.

### Ethics statement

The study protocol was approved by the Ethics Committee of the Second Hospital of Hebei Medical University, and the study was conducted in accordance with the Declaration of Helsinki and Good Clinical Practice guidelines. Written informed consents were provided by all study participants.

### Randomization, grouping and interventions

**Fig. 1:**
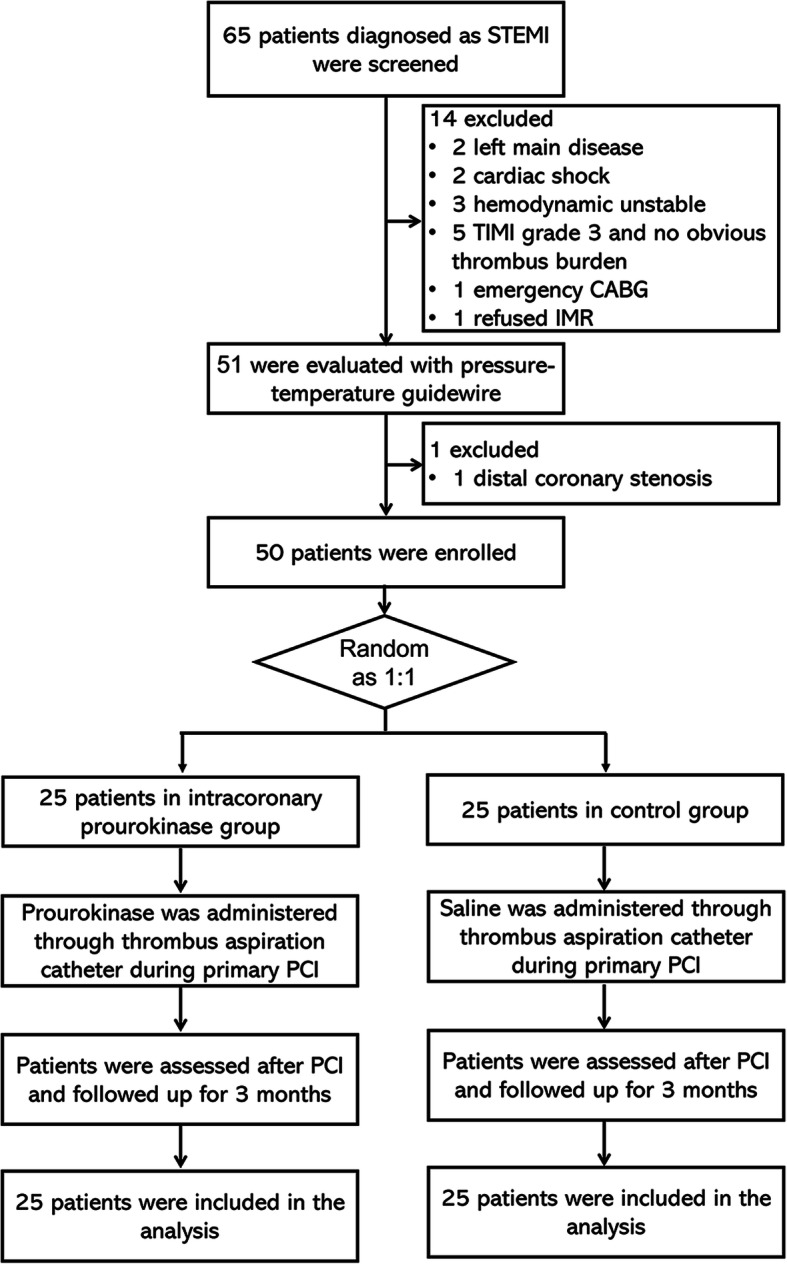
Study flow

Before primary PCI, all the eligible patients were randomly assigned to the intracoronary prourokinase group (*N* = 25) or control group (*N* = 25) as a 1:1 ratio, with a block randomization method. Random sequence was created by SAS 9.2 (SAS Institute, Inc., Cary, North Carolina, USA) software, and the assignment of patients was performed by a nurse who was not involved in the subsequent study. During the primary PCI, for patients in the intracoronary prourokinase group, 10 mg prourokinase (Shanghai Tian Shi Li Pharmaceutical Co, Ltd., Shanghai, China) were administered to the infarction related artery (IRA) through the thrombus aspiration catheter; as for the patients in the control group, 10 ml saline was injected into the IRA by the thrombus aspiration catheter. In addition, the study flow of this study was presented in Fig. 1.

### Primary PCI procedures

After admission, all patients were administered with 300 mg aspirin and 180 mg ticagrelor. Angiotensin-converting enzyme inhibitors or angiotensin receptor blockers were administered to patients who had a systolic blood pressure (SBP) > 120 mmHg, and ß-blocker were given to patients with heart rate (HR) > 60 bpm or there was not wet rale in the auscultation of the lungs. Moreover, statins were given to all patients. All patients were given an initial bolus of 5000 U of intravenous heparin or weight-based dosage according to the discretion of the operator, with additional dose to keep the act time up to 250 ~ 300 s. Tirofiban was administered according to the discretion of the operator as well. Coronary angiography was performed for all patients. All patients were punctured through the forearm approach (radial artery or ulnar artery). A 6F coronary guiding catheter was used to engage the culprit coronary artery, the pressure-temperature guide wire was calibrated in vitro, then was sent to the ostium of the coronary artery to be calibrated inside, which made the distal arterial pressure (Pd) equal to the proximal aortic pressure (Pa). Then the wire was sent to pass across the stenosis into the target distal third of the vessel, after that, the Export AP thrombus catheter (Medtronic Cardiovascular, Santa Rosa, California, USA) was used for thrombus aspiration (repeated 3–5 times). Then the thrombus aspiration catheter was sent to the distal of the lesion. For intracoronary prourokinase group, through the thrombus aspiration catheter, prourokinase (10 mg with saline diluted into 10 ml) was slowly administered until the end of the catheter left the proximal of the occluded lesion. And in the control group, 10 ml saline was injected into the IRA by the thrombus aspiration catheter. Finally, stent implantation was performed when patients’ extent of vascular stenosis was more than 75%. All procedural characteristics were recorded in detail, and thrombus grade was sorted according to the thrombolysis in myocardial infarction (TIMI) criteria as follows [9]: grade 0, no thrombus present; grade 1, possible thrombus present with angiographic characteristics of reduced contrast density, grade 2, explicit thrombus with dimension ≤1/2 artery diameter; grade 3, thrombus with the linear dimension> 1/2, but less than two artery diameter; grade 4, thrombus with dimension ≥2 artery dimension; grade 5, total occlusion.

### Coronary physiological measurements

Coronary physiological indexes including the index of IMR, CFR and FFR were measured after primary PCI. Briefly, 3 ml normal saline was injected into coronary artery through the guide catheter at room temperature, and if the temperature decreased less than 2 degrees, the conduction time of the baseline (T_mn_) was measured, which was repeated 3 times to produce three reproducible and consistent thermodilution curves. If the difference between one of the T_mn_ and the other two measurements was above 30%, it was performed again. The average of the three T_mn_ was taken as the mean baseline transit time (bT_mn_). In addition, Adenosine Disodium Triphosphate (Guangdong Nan Guo Pharmaceutical Co, Ltd., Zhanjiang, Guangdong, China) was given to induce the maximum hyperemia status of microcirculation through the median vein of the elbow, which was confirmed by the decrease of 15 mmHg as the SBP. Another three thermodilution curves were produced and the average conduction time was hyperemic T_mn_ (hT_mn_). The values of IMR, CFR and FFR were measured by the formulas as follows: IMR = Pd*hT_mn_, CFR = bT_mn_/hT_mn_, FFR = Pd/Pa. The catheter and guidewire sensor location remained in the same position to avoid deviation in the measurement of transit time.

### Post-PCI angiographic assessments

Through the coronary angiography post-primary PCI, the TIMI [10], corrected TIMI frame count (CTFC) [11] and TIMI myocardial perfusion grade (TMPG) [12] were evaluated by two experienced intervention cardiologists blinded to the assignment of the patients.

### Myocardial infarct size and myocardial reperfusion assessment

Venous blood was collected every 6 h from all patients after hospital admission, and myocardial injury markers including creatine kinase (CK), creatine kinase isoenzyme-MB (CKMB) and cardiac troponin I (cTnI) were measured, and the peak values of markers were recorded to estimate the infarct size. Besides, all patients were reviewed 12-lead ECG every 10 min within 60 min after primary PCI. A decrease in the sum ST-segment elevation by ≥70% was defined as complete ST-segment resolution (STR) and was regarded as assessment for myocardial reperfusion after primary PCI.

### Cardiac function evaluation

ECG was performed during hospital admission (within 24 h) and repeated at the 3rd month after primary PCI to assess left ventricular ejection fraction (LVEF), left ventricular end-diastolic diameter (LVEDd) and wall motion score index (WMSI). Patients were received two-dimensional ventricular volumes, and LVEF were measured using the modified Simpson’s rule. And the WMS was measured using a 16-segments model by two cardiologists, each segment was visually analyzed and scored from 1 to 5 (1 = normal, 2 = hypokinetic, 3 = akinetic, 4 = dyskinetic, 5 = aneurysm). WMSI = the total score of each segment score /total number of participating integrals.

### Follow up

After discharge from hospital, all patients were followed up monthly for 3 months by outpatient clinic visiting or by telephone and/or medical questionnaires. The major adverse coronary events (MACEs) and hemorrhagic complications were documented from hospital admission to 3 months post-primary PCI. MACEs included cardiac death, various degrees of heart failure, malignant arrhythmia, new myocardial infarction and target vessel revascularization (TVR).

### Study endpoints

The primary endpoints were IMR, CFR and FFR after PCI, and the secondary endpoints included (1) post-PCI TIMI grade, CTFC, TMPG, peak values of CK, CK-MB and cTnI, and percentage of complete STR, (2) LVEF, LVEDd and WMSI within 24 h after hospital admission and at the 3rd month post PCI, (3) hemorrhagic complications and MACEs occurred from hospital admission to 3 months post PCI.

### Statistical analysis

The sample size of this study was calculated based on previous studies [13, 14]. Assuming the IMR in the intracoronary prourokinase group was 30 U (SD = 10) and 40 U (SD = 13) in the control group, with a power of 90%, a two-sided 5% level of significance (α) and a sample size ratio of 1:1, required a sample size of 20 participants in the intracoronary prourokinase group and 20 in control group. Considering a 20% attrition rate, the sample size was inflated to 50 with 25 participants in each group. Continuous variables were presented as mean with standard deviation (SD) if normally distributed or median and interquartile range (IQR) if non-normally distributed. Normality of continuous variable was determined by the Kolmogorov-Smirnov test. Categorical variables were expressed as absolute values and percentage. Differences between two groups were determined by Student’s t-test (for normal distribution values), Mann-Whitney U test (for non-normal distribution values) or Chi-squared test or Fisher’s exact test (for categorical values as appropriate.) Two-sided *P*-value < 0.05 was considered as statistically significant. SPSS 23.0 (SPSS Inc., Chicago, IL, USA) was used for statistical analysis, and GraphPad Prism 7.02 (GraphPad Software Inc., San Diego, CA, USA) was applied to plot graphs.

## Results

### Patients’ characteristics at baseline

No difference of demographic or clinical characteristics was found between intracoronary prourokinase group and control group (Table 1). The values of mean ages were 59.48 ± 14.36 years and 60.96 ± 12.56 years (*P* = 0.700), and the numbers of the male were 21 (80.8%) and 22 (88.0%) (*P* = 0.440) in the intracoronary prourokinase group and control group, respectively. Besides, patients had a mean BMI of 25.69 ± 2.94 kg/m^2^ in intracoronary prourokinase group, and 25.45 ± 2.89 kg/m^2^ in control group (*P* = 769). In addition, the mean levels of SBP were 130.36 ± 24.31 mmHg and 131.72 ± 24.93 mmHg (*P* = 0.846), and the mean DBPs were 77.76 ± 15.56 mmHg and 81.92 ± 18.13 mmHg (*P* = 0.388) in the two groups, respectively. The mean GFR was 91.17 ± 26.26 ml/min in intracoronary prourokinase group and was 96.62 ± 20.49 ml/min in control group (*P* = 0.418). The information of other clinical characteristics was listed in Table 1.

**Table 1:** Baseline characteristics

Parameters	Intracoronary prourokinase group (*N* = 25)	Control group (*N* = 25)	*P* value
Age, mean ± SD, years	59.48 ± 14.36	60.96 ± 12.56	0.700
Male, No. (%)	21 (80.8)	22 (88.0)	0.440
BMI, mean ± SD, kg/m^2^	25.69 ± 2.94	25.45 ± 2.89	0.769
Current smoker, No. (%)	12 (48.0)	13 (52.0)	0.777
Angina history, No. (%)	13 (52.0)	11 (44.0)	0.571
Hypertension, No. (%)	17 (47.2)	19 (52.8)	0.529
Diabetes mellitus, No. (%)	8 (32.0)	6 (24.0)	0.528
Dyslipidemia, No. (%)	8 (30.0)	5 (20.0)	0.333
Heart rate, mean ± SD, bpm	73.24 ± 14.22	77.40 ± 16.32	0.341
SBP, mean ± SD, mmHg	130.36 ± 24.31	131.72 ± 24.93	0.846
DBP, mean ± SD, mmHg	77.76 ± 15.56	81.92 ± 18.13	0.388
Killip grade, No. (%)			0.465
I	10 (40.0)	14 (56.0)	
II	11 (44.0)	9 (36.0)	
III	4 (16.0)	2 (8.0)	
Creatinine, mean ± SD, μmoI/L	85.32 ± 28.85	79.56 ± 16.23	0.389
GRF, mean ± SD, ml/min	91.17 ± 26.26	96.62 ± 20.49	0.418
RBC, mean ± SD, 10^9/L	4.54 ± 0.52	4.51 ± 0.55	0.845
NLR, mean ± SD	8.20 ± 5.53	8.19 ± 6.94	0.996
PLT, mean ± SD, 10^9/L	191.53 ± 91.73	220.92 ± 46.34	0.159
MPV, mean ± SD, fL	7.70 ± 2.70	8.43 ± 1.89	0.222
Medications prior to PCI, No.(%)			
Aspirin	25 (100.0)	25 (100.0)	1.000
Ticagrelor	25 (100.0)	25 (100.0)	1.000
Statins	25 (100.0)	25 (100.0)	1.000
Heparin	25 (100.0)	25 (100.0)	1.000
IIb/IIIa inhibitor	18 (72.0)	14 (56.0)	0.239
Intravenous nitroglycerin	15 (60.0)	18 (72.0)	0.370
Medications at discharge, No.(%)			
ACEI	16 (64.0)	20 (80.0)	0.208
B-blocker	14 (56.0)	18 (72.0)	0.239
Statins	25 (100.0)	25 (100.0)	1.000
DAPT	25 (100.0)	25 (100.0)	1.000

Differences between two groups were determined by Student’s t-test, Mann-Whitney U test, Chi-squared test or Fisher’s exact test, as appropriate. *SD* standard deviation, *BMI* body mass index, *SBP* systolic blood pressure, *DBP* diastolic blood pressure, *GRF* glomerular filtration rate, *RBC* red blood count, *NLR* neutrophiltolymphocyte ratio, *PLT* platelet count, *MPV* mean platelet volume, *PCI* percutaneous coronary intervention, *ACEI* angiotension-converting enzyme, *DAPT* dual anti-platelet therapy

### Patients’ angiographic and procedural characteristics

The angiographic and procedural characteristics were recorded as well, and no difference of angiographic and procedural characteristics was found between the two groups (Table 2). In detail, the number of patients who had infarction of LAD, LCX and RCA were 8 (32.0%), 3 (12.0%) and 14 (56.0%) in intracoronary prourokinase group, and were 10 (40.0%), 3 (12.0%) as well as 12 (48.0%) in control group (*P* = 0.829). There were 4 (16.0%) patients with thrombus grade 1, 2 (8.0%) patients with grade 4, and 19 (76.0%) patients with grade 5 in intracoronary prourokinase group, while there were 1 (4.0%) patient with grade 2, 3 (12.0%) patients with grade 3, 4 (16.0%) patients with grade 4 as well as 17 (68.0%) patients with grade 5 in control group (*P* = 0.750). The information of other angiographic and procedural characteristics was presented in Table 2.

**Table 2:** Angiographic and procedural characteristics

Parameters	Intracoronary prourokinase group (*N* = 25)	Control group (*N* = 25)	*P* value
Infarction related artery, No. (%)			0.829
LAD	8 (32.0)	10 (40.0)	
LCX	3 (12.0)	3 (12.0)	
RCA	14 (56.0)	12 (48.0)	
No. of diseased vessels, No. (%)			0.776
1	4 (16.0)	3 (12.0)	
2	5 (20.0)	7 (28.0)	
3	16 (64.0)	15 (60.0)	
Thrombus grade before PCI, No. (%)			0.750
1	4 (16.0)	0 (0.0)	
2	0 (0.0)	1 (4.0)	
3	0 (0.0)	3 (12.0)	
4	2 (8.0)	4 (16.0)	
5	19 (76.0)	17 (68.0)	
Collateral vessel, No. (%)	15 (60.0)	17 (68.0)	0.556
Temporary pacemaker, No. (%)	2 (8.0)	3 (12.0)	0.637
Number of stents, mean ± SD	1.12 ± 0.33	1.08 ± 0.28	0.646
Stent diameter, mean ± SD, mm	3.01 ± 0.51	3.09 ± 0.55	0.590
Stent length, mean ± SD, mm	23.48 ± 4.49	22.84 ± 6.57	0.690
Stent release pressure, mean ± SD, atm	14.12 ± 3.59	12.72 ± 4.34	0.220
Number of inflations, mean ± SD	3.96 ± 1.54	4.79 ± 2.23	0.134
Pre-dilation pressure, mean ± SD, atm	12.16 ± 3.05	11.48 ± 2.69	0.408
Post dilation, No.(%)	15 (60)	13 (52)	0.569
Post-dilation times, median (IQR)	3 (1–4)	3 (2–5)	0.918
Trans-radial or ulnar artery, No.(%)	25 (100.0)	25 (100.0)	1.000

Differences between two groups were determined by Student’s t-test, Mann-Whitney U test, Chi-squared test or Fisher’s exact test, as appropriate. *FMC* first medical contact, *LAD* left anterior descending, *LCX* left anterior descending, *RCA* right coronary artery, *PCI* percutaneous coronary intervention, *SD* standard deviation, *IQR* interquartile range

### Outcomes of coronary physiology

**Fig. 2:**
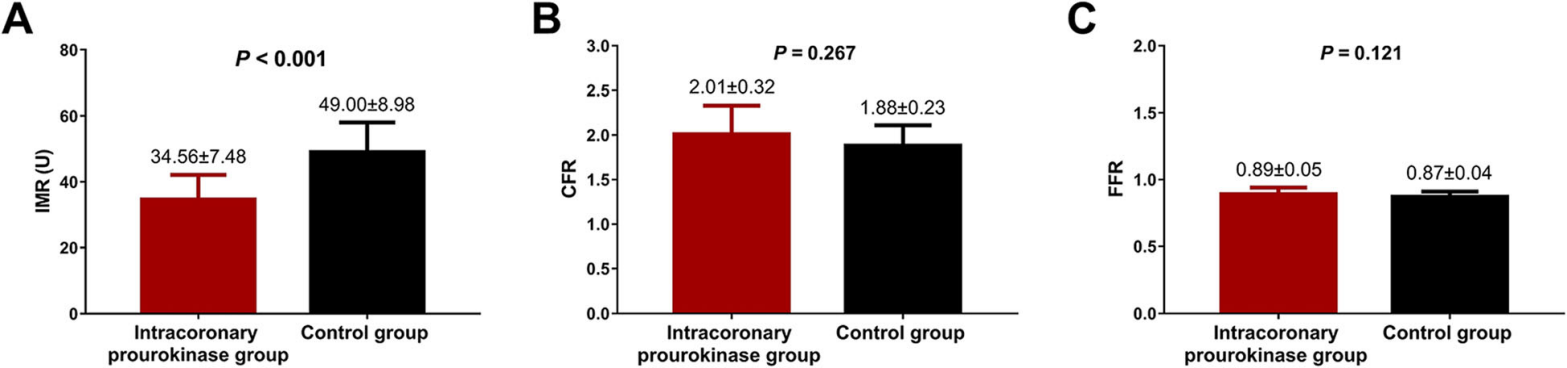
Coronary physiology assessments results. The IMR in intracoronary prourokinase group was reduced compared with control group (**a**), while no difference of CFR (**b**) or FFR (**c**) was found between the two groups. Comparison between the two groups was determined by t-test. *P*-value < 0.05 was considered as statistically significant. IMR, index of microcirculatory resistance; CFR, coronary flow reserve; FFR, fractional flow reserve

The mean value of IMR was markedly decreased in intracoronary prourokinase group than that in control group (34.56 ± 7.48 vs. 49.00 ± 8.98, *P* < 0.001) (Fig. 2a), however, the mean CFR (2.01 ± 0.32 vs. 1.88 ± 0.23, *P* = 0.267) (Fig. 2b) and mean FFR (0.89 ± 0.05 vs. 0.87 ± 0.04, *P* = 0.121) (Fig. 2c) were of no difference between the two groups.

### Outcomes of angiographic assessment, myocardial infarct size and myocardial reperfusion

There were more patients with higher grades of TMPG in intracoronary prourokinase group compared with control group (*P* = 0.024) (Table 3). In addition, the mean CK peak value (*P* = 0.028), CK-MB peak value (*P* = 0.016) and cTnI peak value (*P* = 0.032) were all reduced in intracoronary prourokinase group compared with control group. Moreover, the percentage of patients who had complete STR was increased in intracoronary prourokinase group than that in control group (*P* = 0.005). However, no difference of TIMI grade (*P* = 0.440) or CTFC (*P* = 0.235) was found between the two groups.

**Table 3:** Angiographic outcomes, myocardial infarct size and myocardial reperfusion post PCI

Parameters	Intracoronary prourokinase group (*N* = 25)	Control group (*N* = 25)	*P* value
TIMI grade, No. (%)			0.440
0	0 (0.0)	0 (0.0)	
1	0 (0.0)	0 (0.0)	
2	3 (12.0)	5 (20.0)	
3	22 (88.0)	20 (80.0)	
TMPG, No. (%)			0.024
0	0 (0.0)	0 (0.0)	
1	0 (0.0)	1 (4.0)	
2	4 (16.0)	14 (56.0)	
3	21 (84.0)	10 (40.0)	
CTFC, mean ± SD, frames	16.68 ± 7.26	19.05 ± 6.69	0.235
CK peak value, mean ± SD, U/L	1159.12 ± 617.89	1661.18 ± 1079.60	0.028
CK-MB peak value, mean ± SD, U/L	123.10 ± 31.09	181.56 ± 112.85	0.016
cTnI peak value, mean ± SD, ng/L	38.83 ± 12.53	51.71 ± 26.32	0.032
Complete STR, No. (%)	24 (96.0)	16 (64.0)	0.005

Differences between two groups were determined by Student’s t-test, Mann-Whitney U test, Chi-squared test or Fisher’s exact test, as appropriate. *TIMI* thrombolysis in myocardial infarction, *TMPG* TIMI myocardial perfusion grade, *CTFC* corrected TIMI frame count, *SD* standard deviation, *CK* creatine kinase, *CK-MB* creatine kinase isoenzyme-MB, *STR* ST segment resolution

### Outcomes of cardiac function

The mean values of 1-day LVEF (*P* = 0.899), LVEDd (*P* = 0.938) and WMSI (*P* = 0.724) post PCI were similar between intracoronary prourokinase group and control group (Table 4). However, post PCI, the mean 3-month LVEF was elevated (*P* = 0.017), while LVEDd (*P* = 0.013) and WMSI (*P* = 0.015) were decreased intracoronary prourokinase group compared with control group.

**Table 4:** Cardiac function

	Intracoronary prourokinase group (*N* = 25)	Control group (*N* = 25)	*P* value
1-day LVEF, mean ± SD, %	55.81 ± 5.86	55.60 ± 5.69	0.899
1-day LVEDd, mean ± SD, cm	4.80 ± 0.29	4.81 ± 0.32	0.938
1-day WMSI, mean ± SD	2.12 ± 0.23	2.14 ± 0.24	0.724
3-month LVEF, mean ± SD, %	60.84 ± 2.29	59.18 ± 2.44	0.017
3-month LVEDd, mean ± SD, cm	4.73 ± 0.19	4.89 ± 0.25	0.013
3-month WMSI, mean ± SD	1.84 ± 0.16	1.95 ± 0.15	0.015

Differences between two groups were determined by Student’s t-test. *LVEF* left ventricular ejection fraction, *LVEDd* left ventricular end-diastolic diameters, *WMSI* wall motion score index

### Hemorrhagic complications and MACEs

Post PCI, no case of intracranial hemorrhage, hematemesis, hemoptysis, hematuria, hemorrhage of puncture site was found in both intracoronary prourokinase group and control group (Table 5). As for the other hemorrhagic complications that were recorded, no difference regarding the number of patients who had dermatorrhagia (*P* = 1.000) or gingival bleeding (*P* = 1.000) was found between the two groups. In detail, there were 1 (4.0%) patient with dermatorrhagia and 2 (8.0%) patients with gingival bleeding in intracoronary prourokinase group, and in control group, there was 1 (4.0%) patient who had gingival bleeding. With respect to MACEs post PCI, no difference of the number of total MACEs was found between the tw o groups (*P* = 0.157). Additionally, no cases of cardiac death, TVR or stroke were found in the two groups, and the percentage of total MACE (*P* = 0.157), patients who had stent thrombosis (*P* = 1.000), heart failure (*P* = 0.667) and malignant arrhythmia (*P* = 0.552) were similar between the two groups (Table 6).

**Table 5:** Hemorrhagic complications during 3-month follow up

Items	Intracoronary prourokinase group (*N* = 25)	Control group (*N* = 25)	*P* value
Intracranial hemorrhage, No. (%)	0 (0.0)	0 (0.0)	–
Hematemesis, No. (%)	0 (0.0)	0 (0.0)	–
Hemoptysis, No. (%)	0 (0.0)	0 (0.0)	–
Hematuria, No. (%)	0 (0.0)	0 (0.0)	–
Dermatorrhagia, No. (%)	1 (4.0)	0 (0.0)	1.000
Hemorrhage of puncture site, No. (%)	0 (0.0)	0 (0.0)	–
Gingival bleeding, No. (%)	2 (8.0)	1 (4.0)	1.000

Differences between two groups were determined by Fisher’s exact test

**Table 6:** MACE during 3-month follow up

Items	Intracoronary prourokinase group (*N* = 25)	Control groups (*N* = 25)	*P* value
Total MACE, No. (%)	3 (12.0)	7 (28.0)	0.157
Cardiac death, No. (%)	0 (0.0)	0 (0.0)	–
Stent thrombosis, No. (%)	0 (0.0)	1 (4.0)	1.000
TVR, No. (%)	0 (0.0)	0 (0.0)	–
Heart failure, No. (%)	2 (8.0)	4 (16.0)	0.667
Malignant arrhythmia, No. (%)	1 (4.0)	2 (8.0)	0.552
Stroke, No. (%)	0 (0.0)	0 (0.0)	–

Differences between two groups were determined by Fisher’s exact test. *MACE* major adverse cardiac events, *TVR* target vessel revascularization

## Discussion

There remains several problems to be resolved in STEMI treatment, including the choice of performing PCI only on the culprit artery or in multiple arteries, and the reduction of time delay before PCI treatment, among which the latter is more urgent [15]. Thus, boosting efficacy and safety of PCI in STEMI is constantly a major concern in clinical practice. In addition, the use of prourokinase in myocardial infarction including STEMI has been tested in various clinical trials and studies, which altogether present a good efficacy and safety of prourokinase as a thrombolytic agent [16, 17]. Therefore, we performed this randomized clinical trial which evaluated the efficacy and safety of intracoronary prourokinase during PCI for STEMI treatment, and the main findings were as follows: 1) IMR was decreased in intracoronary prourokinase group compared with control group; 2) TMPG, CK peak value, CK-MB peak value, cTnI and STR were more satisfactory in intracoronary prourokinase group than those in control group; 3) the cardiac function including LVEF, LVEDd and WMSI were better in intracoronary prourokinase group compared with control group; 4) no difference regarding hemorrhagic AEs and MACE incidences was found between the two groups.

The evidence of prourokinase in treating STEMI is still lacking, there are even insufficient studies regarding the prourokinase in treating myocardial infarction. A recent phase IV clinical trial evaluating efficacy and tolerance of prourokinase injection in STEMI patients reveals a successful reperfusion rate of 85.4% in patients, which indicates a good efficacy [6]. And in the study conducted in STEMI patients who receive intracoronary administration of prourokinase through balloon catheter during primary PCI illuminates that the proportions of patients who achieve complete ST-segment resolution, reduced levels of serum CK, CK-MB and TnI are higher than those in patients treated with PCI alone [16]. Another randomized controlled trial reveals more cases of opened infarct-related artery on arrival in the catheterization laboratory and better TIMI frame count in patients receiving PCI with prourokinase than patients receiving only PCI; in addition, at 1-year follow-up, this study also elucidates that patients receiving PCI with prourokinase present with a trend of less incidence of MACE and re-admission to hospital compared with patients treated by PCI alone [17]. In this randomized controlled study, we found that the STEMI patients receiving intracoronary prourokinase during PCI presented with better improvement of IMR compared with patients receiving only PCI, and the TMPG, CK peak value, CK-MB peak value, cTnI, STR as well as the 3-month LVEF, LVEDd and WMSI were also better compared with patients who received only PCI, which were in accordance with the previous trials. And here are several explanations to our findings: the benefits in intracoronary prourokinase group might be derived from that prourokinase is a highly effective fibrin-specific thrombolytic agent, which enhanced the patent efficiency in STEMI patients and thus improved the occlusion of coronary artery compared with only using saline in PCI, therefore the reperfusion of the cardiac muscle was universally more efficient in intracoronary prourokinase group than that in control group, resulting in a better angiographic outcomes, myocardial infarct size reduction, myocardial reperfusion and cardiac function recovery in STEMI patients. In addition, the results in our study also revealed that there was no difference regarding CFR and FFR between the two groups, while, the IMR was notable distinct between the two groups. This result could be caused by the following reasons: first, the sample size in our study was relatively small, which reduced the statistical power to some extent, and subsequently resulted in that the levels of FFR and CFR were numerically but not significantly different between the two groups. Second, the function indicated by the three indexes were distinctive, IMR mainly indicated the microcirculatory function, FFR predominantly indicated the function of the epicardial vascular, while CFR indicated the overall function of coronary artery.

Cardiovascular and hemorrhagic complications are the most common adverse events post PCI treatment, what’s more important is that they are also fatal complications in STEMI patients, for instance, the atrial fibrillation, heart failure and stroke caused by intracranial hemorrhage [18–23]. In a previous clinical trial, the proportions of deaths, post-infarction angina, re-infarction/reocclusion and emergency revascularization were 4.48, 15.2, 4.21, and 8.27% in STEMI patients treated by prourokinase during PCI [6]. In another randomized clinical trial, there is no differences of hemorrhagic complications and MACE incidences between patients treated by prourokinase during PCI and patients treated by only PCI, which is in accordance with our results [16]. A multicenter randomized clinical trial elucidates that although not statistically significant, there seems to be less MACEs and readmissions in patients treated with prourokinase during PCI compared with patients receiving PCI alone [17]. In this trial, we did not find any difference between the two groups with respect to the incidence of hemorrhagic complications or MACEs, which indicated that the use of prourokinase in PCI for STEMI patients did not lead to extra complications compared with a single PCI.

Limitations existed in this trial, which included: 1) the follow up time was relatively short to observe the long-term efficacy and safety of intracoronary prourokinase during PCI in STEMI patients; 2) the study design of a non-blinded method of this trial might cause observer bias; 3) the sample size of this trial was small, which may reduce the statistical power; 4) the level of IMR at baseline was not recorded, which resulted in that the change of microvascular function assessed by IMR from baseline to follow ups could not be evaluated. Therefore, a single/double/triple-blinded clinical trial with a larger sample size, prolonged follow up duration and assessment of baseline microvascular function is needed in the future.

## Conclusions

In conclusion, intracoronary prourokinase during PCI is more efficient and equally tolerant compared with PCI alone in treating STEMI patients.

## Data Availability

All data generated or analysed during this study are included in this published article.

## Abbreviations

STEMI: ST-segment elevation myocardial infarction
PCI: Percutaneous coronary intervention
MACEs: Major adverse coronary events
IMR: Index of microcirculatory resistance
CFR: Coronary flow reserve
FFR: Fractional flow reserve
TMPG: Thrombolysis in myocardial infarction myocardial perfusion grade
CK: Creatine kinase
CK-MB: CK isoenzyme-MB
STR: ST-segment resolution
LVEF: Left ventricular ejection fraction
WMSI: Wall motion score index
LVEDd: Left ventricular end-diastolic diameter
ECG: Electrocardiograph
SBP: Systolic blood pressure
HR: Heart rate
TIMI: Thrombolysis in myocardial infarction
CTFC: Corrected TIMI frame count
TMPG: TIMI myocardial perfusion grade
TVR: Target vessel revascularization
SD: Standard deviation
IQR: Interquartile range
